# Commentary: Acute Myocardial Response to Stretch: What We (don't) Know

**DOI:** 10.3389/fphys.2017.00121

**Published:** 2017-03-02

**Authors:** Reza Vahidi, Siyavash Joukar

**Affiliations:** ^1^Department of Veterinary Sciences, Baft Branch, Islamic Azad UniversityBaft, Iran; ^2^Cardiovascular Research Center, Institute of Basic and Clinical Physiology Sciences, Kerman University of Medical SciencesKerman, Iran; ^3^Department of Physiology and Pharmacology, School of Medicine, Kerman University of Medical SciencesKerman, Iran; ^4^Physiology Research Center, Institute of Neuropharmacology, Kerman University of Medical SciencesKerman, Iran

**Keywords:** myocardial stretch, apelinergic system, frank starling mechanism, slow force response, inotropic effect

Recently, Neves and colleagues reviewed some of the factors that orchestrate acute myocardial response to stretch (Neves et al., 2015). We would like to compliment the authors for their interesting work. They addressed the most extensively studied pathways for hemodynamic stress alleviation. According to their comments, well-characterized mechanisms, Frank-Starling Mechanism (FSM) and Slow Force Response (SFR) are contributed in the systolic adaptation of heart (Kockskämper et al., 2008; Neves et al., 2015). However, they have not paid attention to the role of apelinergic system as a relatively new player which can be involved in the SFR mechanism. Decreased heart contractility in apelin gene-deficient mice associated with pressure overload and aging and also significant lower of the cardiomyocyte Frank-Starling gain in apelin receptor-knockout mice are evidences in favor of involvement of apelinergic system in cardiac contractility aspects (Kuba et al., 2007). This system can couple to the angiotensin receptor like-1 (APJ), an orphan G protein-coupled receptor, and it is able to function as a bimodal switch. Based on the applied stimulus, it can translate different chemical (apelin) and mechanical (stretch) signals into opposite phenotypical behavior by modulating the levels of activation of G-protein signaling vs β-arrestin signaling (Scimia et al., 2012; Gerilechaogetu et al., 2014). When mechanical stretch is superimposed on apelin stimulation, the APJ activation interferes with apelin mediated G-protein signaling (G-protein-independent-β-arrestin-dependent signaling pathway). This leads to a pathologic stretch-mediated hypertrophy (apelin-independent effects of APJ) that could be minimizing by administering of apelin or knocking-down beta-arrestins (Scimia et al., 2014). On the other hand, activation of APJ by apelin can regulate cardiac contractility through various signaling mechanisms (Figure 1). Apelin induces a slowly developing but sustained myocardial contraction (Slow Force Response) at sub-nanomolar concentrations and engages in the maintaining of intercellular communication and electrophysiological properties of cardiac tissue (Szokodi et al., 2002; Farkasfalvi et al., 2007; Scimia et al., 2014). In line with these reports, acute apelin infusion increases the cardiac output and load-independent measures of myocardial contractility (such as ventricular elastance and preload recruitable stroke work) whereas chronic administration exerts an increase in the cardiac output without inducing left ventricular hypertrophy (Japp and Newby, 2008; Scimia et al., 2014).

**Figure 1:**
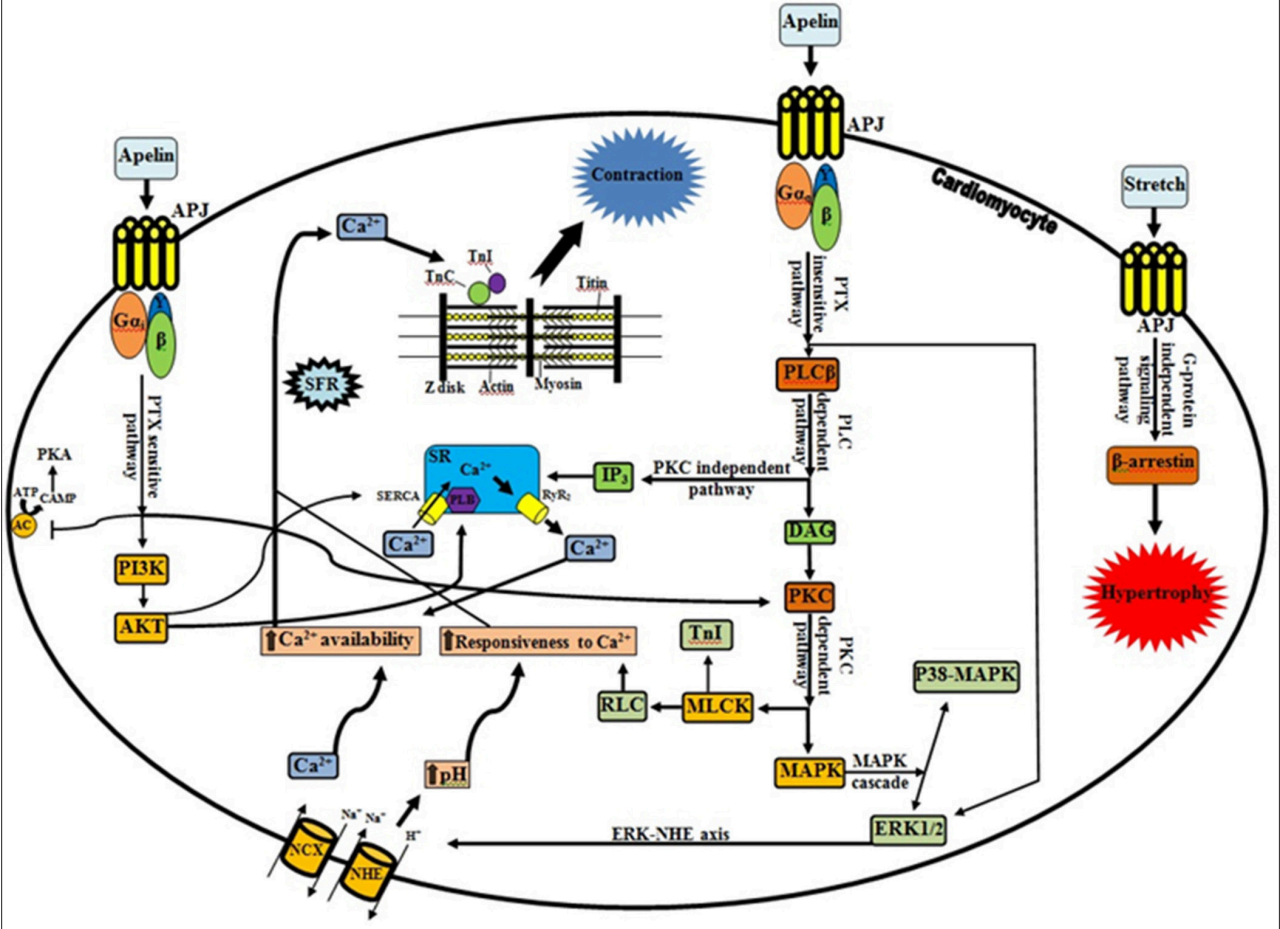
**Apelinergic signaling pathway**. G protein-dependent, including PTX-sensitive and -insensitive pathways; and G protein-independent pathway, mediating the effects of apelin receptor (Kalea and Batlle, 2010; Chen et al., 2014). In contrary to dominant stretch that causes β-arrestin-mediated pathologic hypertrophy, apelin through APJ and G proteins can induce inotropic effects and plays a protective role. There is a functional interplay between apelin and stretch, such that apelin blunts pathological signaling from the stretch and stretch in turn profoundly interferes (via β-arrestins) with G-protein activation relevant to apelin signaling. Progressive increase in Ca^2+^ transient is the central player in the positive inotropic effect of apelin (Wang et al., 2008; Neves et al., 2015). As it shown, apelin through increasing of RyR_2_ channel (Wang et al., 2008), NCX (Szokodi et al., 2002; Japp and Newby, 2008), PLB and SERCA activity (Wang et al., 2008), provide the Ca^2+^ required for contraction. The Ca^2+^ molecules subsequently bind to the contractile proteins such as TnC, which causes interaction of thick and thin filaments (Sussman et al., 2011; Gerilechaogetu et al., 2014). Besides RLC activation by PKC (sustained, fine-tuning regulation of contraction), PKC-mediated phosphorylation of NHE and NCX is essential for the maintenance of the increased contractility and for the prevention of the slow force decline (Perjés et al., 2014; Neves et al., 2015). It is noteworthy that the intracellular signals via PKC-ERK axis and Akt phosphorylation can be downstream elements of PTX-sensitive fashion of apelinergic system. Improving of calcium handling (direct or indirect modification of calcium cycling proteins), increasing of Ca^2+^ loading of SR along with translocation of AKT to the SR (due to increased of PLB phosphorylation), and increasing of the SERCA-PLB activity (via downregulation of phosphatase PP1) are proposed mechanisms for the increased contractility by cardiac specific AKT (Sussman et al., 2011; Gerilechaogetu et al., 2014). Interestingly, Wang et al. demonstrated that SR Ca^2+^ content is decreased by apelin and greater increase of NCX activity than that of SERCA activity, is proposed as an explanation (Wang et al., 2008). APJ, Angiotensin receptor like-1; PLC, phospholipase C; IP3, inositol trisphosphate; SR, sarcoplasmic reticulum; SERCA, sarco/endoplasmic reticulum Ca^2+^ ATPase; PLB, phospholamban; RyR2, ryanodine receptor; DAG, diacylglycerol; PKC, protein kinase C; MLCK, myosin light chain kinase; TnI, troponin I; RLC, myosin regulatory light chain; MAPK, Mitogen-activated protein kinase; ERK1/2, extracellular signal-regulated kinase 1/2; NHE, Na^+^/H^+^ exchanger; NCX, Na^+^/Ca^2+^ exchanger; AC, adenyl cyclase; ATP, adenosine triphosphate; cAMP, cyclic adenosine monophosphate; PKA, protein kinase A; PI3K, phosphoinositide 3-kinase; AKT, protein kinase B; Gi, inhibitory G protein; Gq, phospholipase C-activating G protein; SFR, slow force response; TnC, troponin C; →, Stimulatory effect; ⊣, Inhibitory effect.

Apelin can enhance cardiac contractility via both pertussis toxin (PTX)-sensitive and -insensitive G proteins (Gα_i_ and Gα_q_, respectively) but independent of angiotensin II, endothelin-1, catecholamines, and nitric oxide (Perjés et al., 2014; Scimia et al., 2014). Increasing in phospholipase C activity (especially PLCβ) besides activating inositol trisphosphate (protein kinase C-independent pathway) (Yang et al., 2015) can trigger downstream signaling cascades by induction of phosphoinositide hydrolysis and diacylglycerol-mediated PKC activation (PKC-dependent pathway) (Szokodi et al., 2002; Japp and Newby, 2008). There are accumulating lines of evidence which suggest that the protein kinase C (PKC) activation a crucial messenger for the evoked positive inotropic response of apelin. PKC can phosphorylate the sarcolemmal Na^+^/H^+^ exchanger (NHE), myosin light chain kinase (MLCK) and mitogen-activated protein kinase (MAPK), resulting in a strong positive inotropic effect (Szokodi et al., 2002; Perjés et al., 2014) (Figure 1). Cytoplasmic alkalinization that drives up-regulation of hypertrophic genes and increasing protein synthesis along with subsequent sensitization of cardiac myofilaments to intracellular Ca^2+^ (without altering cytoplasmic Ca^2+^ transients and voltage-gated Ca^2+^ channels) are direct effects of NHE activation (Szokodi et al., 2002; Farkasfalvi et al., 2007; Japp and Newby, 2008). On the other hand, NHE-mediated accumulation of intracellular Na^+^ can indirectly promote a rise in Ca^2+^ concentration within cells via reverse mode Na^+^/Ca^2+^ exchanger (NCX) (Szokodi et al., 2002; Japp and Newby, 2008). Therefore, it is plausible to assume that NHE and NCX are proximal and distal components of a contiguous signaling pathway respectively, and apelin can stimulate myocardial contractility by enhancing myofilament responsiveness to Ca^2+^ (transitory form) along with increase intracellular Ca^2+^ availability (SFR) (Farkasfalvi et al., 2007; Japp and Newby, 2008; Gerilechaogetu et al., 2014; Perjés et al., 2014). In addition, apelin may increase the Ca^2+^-sensitivity of the contractile machinery through cardiac MLCK activation. This Ca^2+^/calmodulin-independent isoform has potential phosphorylation sites for PKC. Therefore, collaboration of PKC-dependent myosin regulatory light chain (RLC) and troponin I (TnI) phosphorylation can more improve the cardiac contractility (Perjés et al., 2014; Neves et al., 2015). Recent studies suggest that PKC and ERK1/2 are parallel and independent signaling pathways mediating the inotropic effect of apelin (Malo et al., 2007; Gerilechaogetu et al., 2014). Moreover, apelin via APJ activation can increase angiotensin converting enzyme 2 (ACE2) promoter activity and ACE2 expression, which improved the cardiac functions independently of AT1R signaling (Sato et al., 2013).

Colocalization of PKCε and Focal Adhesion Kinase (FAK) to costameres and focal adhesions and the relation of FAK activity to various signaling pathways such as G protein-coupled receptors (ERK1/2 and PKCε), raises the possibility that there is a link between apelinergic system and integrin-mediated signaling (Heidkamp et al., 2003). However, additional evaluations will be required to substantiate this possibility.

In conclusion, given a wide array of putative salutary effects of apelinergic system on heart contractile reserve, it can be considered as the other contributor which engages in the cardiac contractility response, especially in the SFR mechanism, to acute hemodynamic overload.

## Author contributions

SJ developed the idea and contributed to preparation of paper. RV contributed to preparation of paper and figure design.

### Conflict of interest statement

The authors declare that the research was conducted in the absence of any commercial or financial relationships that could be construed as a potential conflict of interest.
